# Endo-Aortic Clamp for Minimally Invasive Redo Mitral Valve Surgery: Early Outcome

**DOI:** 10.3390/jcdd11110358

**Published:** 2024-11-06

**Authors:** Cristina Barbero, Marco Pocar, Andrea Costamagna, Cecilia Capozza, Valentina Aloi, Erik Cura Stura, Stefano Salizzoni, Mauro Rinaldi

**Affiliations:** 1 Division of Cardiac Surgery, Cardiovascular and Thoracic Department, Città della Salute e della Scienza, University of Turin, 10126 Turin, Italy; cecicap@hotmail.it (C.C.); valentina.aloi@unito.it (V.A.); cserik87@gmail.com (E.C.S.); stefano.salizzoni@unito.it (S.S.); mauro.rinaldi@unito.it (M.R.); 2 Intensive Cardiac Care Unit, Anesthesia Intensive Care and Emergency Department, Città della Salute e della Scienza, University of Turin, 10126 Turin, Italy; andrea.costamagna@hotmail.it

**Keywords:** mitral valve, adhesions, re-sternotomy, re-exploration, bleeding, reoperation, minimally invasive cardiac surgery

## Abstract

Objective: Redo mitral valve surgery still represents a challenging and high-risk procedure in cardiac surgery. The incidence of cardiac structural injuries during re-sternotomy remains consistent and is reported to be an independent risk factor for hospital mortality. Minimally invasive cardiac surgery with retrograde femoral arterial perfusion and endo-aortic clamping avoids re-entry injuries and reduces the requirement for dissection of adhesions and the risk of damage to cardiac structures. The aim of this study is to analyze redo patients undergoing mitral valve surgery with retrograde arterial perfusion and endo-aortic clamping setting. Methods: A retrospective analysis was performed on patients undergoing surgery from 2006 to 2022. Exclusion criteria were more than mild aortic regurgitation, moderate-to-severe peripheral vascular disease, dilated ascending aorta, and a lack of preoperative vascular screening. The primary outcome was perioperative mortality. Results: Two hundred eighty-five patients were analyzed. Mean age was 63.8 ± 13.3 years, mean EuroSCORE was 16.5 ± 14.5%, and one quarter of the patients had undergone two or more previous procedures via sternotomy. Perioperative mortality was 3.9% (11/285). Stroke was reported in six (2.1%) patients. Median intensive care unit and hospital length of stay were 1 and 8 days, respectively. Conclusions: Endo-aortic clamping setting in redo MV surgery avoids re-entry injuries and allows the surgeon to clamp the aorta and deliver the cardioplegia with minimal dissection of adhesions. In high-volume and experienced centers, this approach can be applied safely and effectively and may in the near future become the standard of care for redo mitral valve surgery.

## 1. Introduction

Redo mitral valve (MV) operations represent a technically challenging and high-risk procedure in cardiac surgery [1,2]. To date, it is still associated with an increased risk of major adverse events and 30-day mortality, and reoperation is an independent predictor of death in most commonly adopted risk models [3,4].

The presence of dense adhesions and the risk of damage to the right ventricle, great vessels, or patent bypass grafts in case of previous coronary surgery may render redo MV operations demanding through a median re-sternotomy. Injuries to cardiac structures are reported in up to 9% of re-sternotomies and are the cause of increased hospital mortality [5].

The right mini-thoracotomy approach abolishes sternal re-entry injuries and reduces the need for extensive and time-consuming dissection of adhesions. In this respect, retrograde arterial perfusion (RAP) through the femoral artery combined with endo-aortic clamping (EAC) allow redo procedures to be carried out in a more straightforward manner. The EAC device consists of a three-lumen catheter with an elastomeric balloon near its tip customized for endoluminal occlusion of the ascending aorta and contemporary delivery of antegrade cardioplegia or aortic root venting.

Experience with this device is scarce in the literature; the main concerns regard the demanding learning curve, the hypothetical increased risk of neurological and vascular complications related to the retrograde perfusion and retrograde catheter manipulation, and the adequateness of myocardial protection under specific conditions, such as prior coronary surgery with patent grafts.

The aim of this study is to depict our 15 years of experience in redo MV surgery with EAC.

## 2. Materials and Methods

### 2.1. Study Design

A retrospective single-center analysis of redo patients undergoing minimally invasive MV surgery with the EAC device was performed. Data were prospectively collected into a dedicated database from January 2006 to December 2022 and approved for use in research by our Institutional Review Board (protocol number 0047596, 29 April 2021).

All the patients enrolled had a history of at least one prior operation performed through median sternotomy and underwent a preoperative vascular screening with computerized tomography scan and/or aortic and ileo-femoral angiography. Exclusion criteria were the following: associated surgical procedures except tricuspid valve surgery, atrial septal defect closure and atrial fibrillation ablation, age < 18 years, more than mild aortic regurgitation, moderate-to-severe peripheral vascular disease, ascending aorta diameter > 40 mm, and lack of preoperative imaging of the aorta and ileo-femoral axes. The primary outcome was perioperative mortality. Secondary outcomes were stroke, re-exploration for bleeding, and intensive care unit (ICU) length of stay.

Perioperative mortality was defined as any death prior to discharge or within 30 days from the operation. Stroke was defined as a new-onset persistent clinical neurologic deficit at the time of hospital discharge and/or the presence of focal cerebral ischemic infarcts detectable by conventional neuroimaging techniques.

### 2.2. Surgical Technique

The right mini-thoracotomy approach with EAC setting routinely used in our clinical practice has been described previously [6,7,8,9]. After full heparinization, peripheral cardiopulmonary bypass (CPB) is established, and the patient is cooled to 30 °C. Arterial cannulation is gained with a 21F or 23F cannula with a sidearm (Edwards Lifesciences, Irvine, CA, USA). In the case of small arteries or arterial perfusion pressure > 300 mmHg during full-flow CPB, a contralateral femoral cannulation is added and connected to the arterial perfusion line in a Y fashion. Venous return is obtained with a double femoral and percutaneous jugular cannulation in all cases.

**Figure 1:**
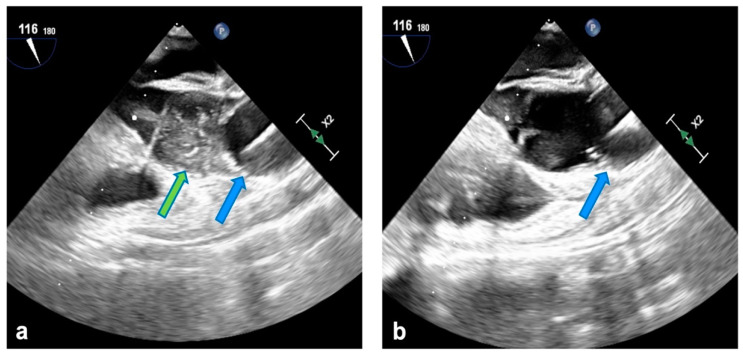
(**a**) Endo-aortic balloon in ascending aorta (blue arrow); cardioplegia delivery (green arrow). (**b**) Correct positioning of the endo-balloon in ascending aorta at the end of cardioplegia administration.

Clamping and cardioplegia delivery are obtained through the balloon catheter (Intraclude^®^, Edwards Lifesciences, Irvine, CA, USA) inserted through the sidearm of the arterial cannula. Antegrade myocardial protection is always provided with crystalloid St. Thomas hospital solution 1 (Plegisol^TM^, Hospira Inc., Lake Forest, IL, USA) or Bretschneider solution (Custodiol^®^ HTK, Raleigh, NC, USA). Adenosine is infused directly in the aortic root to enhance immediate asystole, therefore enabling accurate and steady positioning of the balloon in the ascending aorta (Figure 1a). When feasible, removal of the crystalloid solution through a minimal right atrial incision is preferred to hemofiltration during CPB in patients receiving Bretschneider cardioplegia. Superior and inferior vena cava snaring are obtained by placing tourniquets around the vessels or with endovascular balloons. Continuous monitoring with transesophageal echocardiography is mandatory for proper positioning of the venous cannulae and to assess aortic occlusion and cardioplegia delivery and mitigate the hazards of endo-balloon migration towards the aortic arch or valve (Figure 1b). Furthermore, right radial arterial pressure is continuously monitored to promptly detect distal dislodgement because transesophageal echocardiographic views may prove inadequate in these conditions, delaying diagnosis.

No additional topical cooling nor retrograde cardioplegia through the coronary sinus was used. In most of the cases with patent grafts, a safe asystole was reached after cardioplegia delivery. In few cases of persistent ventricular activity, patients were cooled to 26–28 °C, with no additional intervention on myocardial protection. At the end of the procedure, air is vented through the tip of the cardioplegia line with the balloon inflated.

### 2.3. Data Presentation and Statistics

Categorical variables are reported as frequency (%). Normally and non-normally distributed continuous variables are presented as mean ± standard deviation (SD) or median [interquartile ratio], respectively. Normality of distribution was verified with the Kolmogorov–Smirnov test. Patients who underwent redo mitral surgery via sternotomy were mostly confined to the first years of our experience and to non-elective operations. In parallel, the use of trans-thoracic clamping also was inconstant and partially dictated by the temporary unavailability of the endo-clamp. Thus, comparisons between different approaches were considered futile and beyond the scope of this report.

## 3. Results

**Figure 2:**
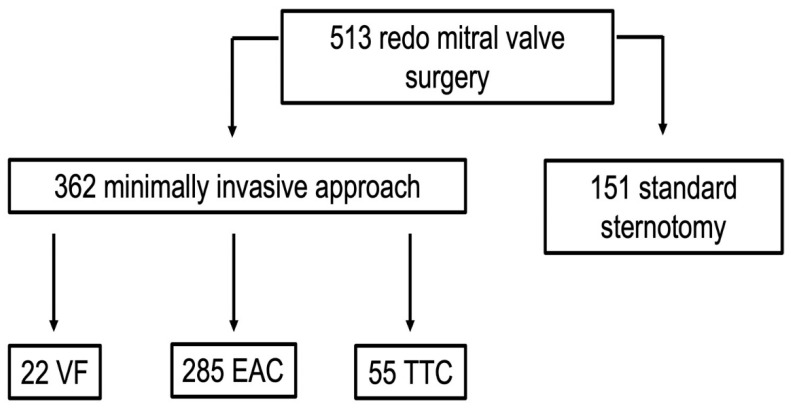
Redo mitral valve surgery during the study period. VF: ventricular fibrillation; EAC: endo-aortic clamp; TTC: trans-thoracic clamp.

During the study period, 513 consecutive redo patients underwent MV surgery at our department (Figure 2). Among the 362 (70.6%) patients who were addressed with a minimally invasive approach via right mini-thoracotomy, 285 (78.7%) met the criteria and were selected for RAP and EAC.

Patients’ characteristics are summarized in Table 1. Mean age was 63.8 years, and 158 (55.4%) patients were female. Mean logistic EuroSCORE was 16.5%, whereas 75 (26.3%) patients had undergone two or more previous cardiac procedures via sternotomy. Patent bypass grafts were present in 32/36 (88.8%) patients with prior coronary operations. MV surgery had been the initial primary operation in 192 (67.4%) cases.

Intraoperative variables and postoperative outcomes are reported in Table 2. Isolated MV surgery was performed in most of the cases (205/285, 71.9%), while a conservative procedure was feasible in 42/193 (21.8%) patients with native valve. The need for an additional contralateral arterial cannula for pressure higher than 300 mmHg during full-flow CPB was reported in 21 (7.4%) patients. Balloon puncture or tear occurred in two (0.7%) cases. In one case, the balloon was repositioned, while in the other one the operation was carried out on the beating heart with deeper hypothermia. Conversion to standard sternotomy was necessary in one patient (0.3%) due to uncontrolled bleeding from the left atrial appendage. No cases of iatrogenic acute aortic dissection were reported.

Perioperative mortality was 3.9% (11/285 patients). Stroke was reported in 6 (2.1%) patients, whereas re-exploration for bleeding was necessary in 20 (7%) cases. Median ICU and hospital length of stay were 1 and 8 days, respectively. No cases of perioperative myocardial infarction were reported.

## 4. Discussion

Re-sternotomy remains a dangerous phase of the operation in patients with previous cardiac surgical procedures [5,10]. Severe injuries to the heart or great vessels during re-sternotomy are still consistently reported and are related to operative death in up to one third of these patients [5,10,11]. Patent coronary bypass grafts may further increase the risks, whereas the presence of a rigid valve prosthesis in the aortic position may impair adequate exposure of the MV and render redo procedures even more cumbersome and challenging through a standard sternotomy approach [2,12].

The minimally invasive setting avoids re-sternotomy and related injuries, reduces the need for extensive and time-consuming dissection of adhesions, enhances a better and direct vision of the MV, and, with the EAC device, allows the surgeon to clamp the aorta and deliver cardioplegia with a sort of no-touch technique.

The present report analyzes data of our more than 15 years of experience in redo patients undergoing minimally invasive MV surgery with the EAC setting. Figure 2 depicts how this strategy has become the standard of care at our institution. Over 70% of all redo patients have undergone surgery through a minimally invasive approach, and almost 80% with the EAC setting. The main exclusion criteria for this approach in our everyday clinical practice remains the diagnosis of moderate-to-severe peripheral vascular disease, which precludes safe RAP and retrograde balloon manipulation.

Retrospective analysis on a cohort of nearly 300 high-risk patients—mean logistic EuroSCORE 16.5%; two or more previous sternotomies in 26% of cases; patent coronary bypass grafts in 13%; prior MV surgery in 67%—overall depicts encouraging early outcomes. Mortality was reported in 11 patients (3.9%), significantly lower than predicted with EuroSCORE. Moreover, major complications are rarer when compared to data recently reported in the literature on redo patients undergoing MV surgery through median re-sternotomy or mini-thoracotomy with different myocardial protection protocols [13,14]. These results may reflect long-standing experience, leading to a rigorous and standardized preoperative screening algorithm on all potential candidates for a minimally invasive approach and a consequently well-established learning curve versus different arterial perfusion and aortic clamping strategies to identify higher risk conditions and indicate the safest approach.

Two thirds (67%) of the patients in this report had undergone previous MV surgery, representing a specifically high-risk subgroup of redo patients with an increasing prevalence in the general and surgical population [15]. Mortality for reoperative MV surgery remains significant even in high-volume centers, with reported rates between 6% and 15% for elective surgery, 17% and 40% in case of third or fourth operations, and around 18% for emergencies [16,17,18,19]. Even the analysis of this subgroup of patients confirmed better than expected results in our experience with a perioperative mortality and stroke rate of 2.8%.

Despite the specific advantages of the right mini-thoracotomy approach in redo MV surgery being well recognized, this strategy is not routinely adopted in this scenario at high-volume heart valve centers worldwide. The main critiques are primarily related to the supposed increased risk of stroke with RAP and retrograde catheter manipulation, which in turn determined the inconstant popularity of EAC. However, studies focused on RAP have outlined a higher rate of stroke only in case of severe peripheral vascular disease, and recent multicenter experiences were able to demonstrate the safety and effectiveness of the EAC setting. A multicenter study focused on right mini-thoracotomy MV surgery with EAC carried out by Casselman et al. showed a stroke rate of only 0.8% (4 of 500 patients) [20]. Similar results were reported by a retrospective analysis from three high-volume centers in Europe comparing the EAC with the TTC [21] and by our group in a report on 460 consecutive patients who underwent right mini-thoracotomy MV surgery with different strategies of aortic clamping and arterial perfusion. Results showed that with a proper preoperative assessment and allocation to the most adequate technique, early mortality and stroke rates were low and comparable between the different strategies adopted [6]. The present study, focused on redo MV patients, outlines similar results with low risk of perioperative adverse events.

Another point of concern when looking at redo patients with prior coronary revascularization and patent grafts is the safety of the minimally invasive approach with EAC in terms of myocardial protection. In this condition, the heart may develop ventricular fibrillation due to partial reperfusion. The strategy adopted in our practice is applying deeper systemic hypothermia, lowering core temperature to 26–28 °C. Conversely, the endo-balloon inflated in the ascending aorta may prevent air embolism, which is a well-known complication in cases of the procedure being performed with a fibrillating heart. This technique also allows the surgeon to maintain a relatively bloodless field throughout the procedure despite patent bypass grafts. In our experience, no cases of perioperative myocardial injury were observed. Moreover, our group has already analyzed outcomes on myocardial protection during minimally invasive MV surgery. A propensity-weighted analysis was performed to compare patients undergoing MV surgery with EAC with patients undergoing MV surgery with TTC: no cases of myocardial infarction or low cardiac-output syndrome were reported overall, and no differences were recorded in the release of myocardial markers, lactates levels, and the need for inotropic support at different time points after surgery, while CK-MB peak levels were significantly lower in the EAC group [22].

Finally, particularly with the first-generation balloon, the learning curve is steep, not only for the surgeon but also for the entire team. The most recent device, the Intraclude catheter, has been designed as an evolution of the previous EAC with reduced size of the catheter, a wider cylindrical shape of the balloon with a better contact surface, and better adhesion to the aortic wall, allowing for more reliable sealing after inflation. Therefore, the incidence of dislocation and/or blood leak in the aortic root is reduced. Moreover, the Intraclude shaft is curve-shaped, allowing better adhesion to the aortic arch and an improved tip orientation towards the aortic valve for cardioplegia delivery [23].

This is a retrospective analysis, and this is an inherent limitation of this study. In addition, the surgeons involved have developed substantial experience in minimally invasive MV surgery and EAC setting, which may possibly correlate with our encouraging results in this high-risk patient population. Therefore, similar outcomes may not be readily reproducible at different institutions when at the beginning of the learning curve with this device. In fact, our attitude has been to expand the mini-thoracotomy philosophy potentially to every candidate for MV surgery. Similarly, EAC has been considered an optimal standard for redo MV operations. The uneven distribution over time and the inconstant availability of the EAC device rendered a comparison with different approaches to be of limited value when applied to our patient population, as stated above (see Section 2.3).

## 5. Conclusions

RAP with EAC setting in minimally invasive redo MV surgery avoids re-entry injuries, allows minimal dissection of adhesions, and gains control of the aorta for a safe clamping and straightforward delivery of cardioplegia. Our experience depicts how lower-than-expected perioperative mortality and major complication rates can also be obtained in high-risk redo patients with routine adoption of this strategy. In experienced, high-volume centers, the right mini-thoracotomy approach with EAC may in the near future be considered the standard of care for reoperative MV surgery.

## Figures and Tables

**Table 1:** Preoperative patients’ characteristics.

	*n* = 285
Female	158 (55.4)
Age, years	63.8 ± 13.3
BMI, kg/m^2^	24.6 ± 4.8
Log EuroSCORE	16.5 ± 14.4
NYHA class ≥ 3	173 (60.7)
Atrial fibrillation	153 (53.7)
Chronic kidney disease	55 (19.3)
Hypertension	177 (62.1)
COPD	31 (10.9)
Cirrhosis	4 (1.4)
Diabetes	48 (16.8)
Mild peripheral vasculopathy	15 (5.3)
Coronary artery disease	20 (7)
Two or more previous cardiac operations	75 (26.3)
Previous CABG	36 (12.6)
Previous MV repair	100 (35.1)
Previous MV replacement	92 (32.3)
Previous tricuspid valve repair	3 (1.1)
Previous aortic valve surgery	39 (13.7)
LVEDD, mm	53.4 ± 13.1
LVEF, %	53.1 ± 11.4
sPAP, mmHg	47.7 ± 16.1

BMI, body mass index; NYHA, New York heart association; COPD, chronic obstructive pulmonary disease; CABG, coronary artery bypass graft; MV: mitral valve; LVEDD, left ventricular end-diastolic diameter; LVEF, left ventricular ejection fraction; sPAP, systolic pulmonary artery pressure. Data are presented as *n* (%) or mean ± SD, as appropriate.

**Table 2:** Intraoperative data and postoperative outcomes.

	*n* = 285
Isolated MV surgery	205 (71.9)
MV repair	42 (14.7)
MV replacement	143 (50.2)
Mitral prosthesis replacement	100 (35.1)
TV surgery	65 (22.8)
ASD closure	10 (2.8)
AF ablation	13 (4.6)
CPB time, min	141.6 ± 45.3
Cross-clamp time, min	93.9 ± 28.6
Acute aortic dissection	–
Conversion to sternotomy	1 (0.3)
Mechanical ventilation > 72 h	29 (10.2)
Re-intubation	21 (7.4)
Acute kidney injury	18 (6.3)
Stroke	6 (2.1)
Re-exploration for bleeding	20 (7)
Thoracic wound dehiscence	5 (1.8)
Permanent pacemaker	18 (6.3)
ICU length of stay, days	1 [1–3]
In-hospital length of stay, days	8 [7–13]
30-day mortality	11 (3.9)

MV, mitral valve; TV, tricuspid valve; ASD, atrial septal defect; AF: atrial fibrillation; CPB, cardiopulmonary bypass; ICU, intensive care unit. Data are presented as *n* (%), mean ± SD or median [IQR], as appropriate.

## Data Availability

The data presented in this study are available upon reasonable request from the corresponding author.
